# Effects of an early multidisciplinary intervention on sickness absence in patients with persistent low back pain—a randomized controlled trial

**DOI:** 10.1186/s12891-022-05807-7

**Published:** 2022-09-10

**Authors:** Annette Fisker, Henning Langberg, Tom Petersen, Ole Steen Mortensen

**Affiliations:** 1 Department of Occupational and Social Medicine, Holbæk Hospital, part of Copenhagen University Hospital, Smedelundsgade 60, 4300 Holbæk, Denmark; 2 Section of Social Medicine, Department of Public Health, University of Copenhagen, Øster Farimagsgade 5, Copenhagen K, Denmark; 3 Back- and Rehabilitation Centre Copenhagen, Mimersgade 41, Copenhagen N, Denmark

**Keywords:** Low back pain, Back pain, Sick leave, Return to work, Occupational health, Multidisciplinary intervention, Rehabilitation, Randomized controlled trial

## Abstract

**Background:**

Multidisciplinary rehabilitation is recommended to reduce sickness absence and disability in patients with subacute or chronic low back pain (LBP).

This study aimed to investigate whether a 12-week coordinated work oriented multidisciplinary rehabilitation intervention was effective on return to work and number of days off work during one-year follow-up when compared to usual care.

**Methods:**

This study is a randomized controlled trial comparing the effectiveness of a 12-week multidisciplinary vocational rehabilitation program in addition to usual treatment. 770 patients with LBP, who were sick-listed, or at risk of being sick-listed were included in the study.

The primary outcome was number of days off work due to LBP. The secondary outcomes were disability, health-related quality of life, pain, psychological distress and fear avoidance behavior.

Data were collected at baseline, at the end of treatment, and at 6- and 12-months follow-up. Analyses were carried out according to the “intention-to-treat” principles.

**Results:**

A significant decrease in the number of patients who were on sick-leave was found in both groups at the end of treatment and at 6- and 12-months follow-up. Additionally, disability, pain, health related quality of life, psychological distress, and fear avoidance beliefs improved in both groups. No statistically significant differences were found between the groups on any of the outcomes.

**Conclusions:**

The coordinated multidisciplinary intervention had no additional effect on sickness absence, disability, pain, or health related quality of life as compared with that of usual care.

**Trial registration:**

This study was retrospectively registered in ClinicalTrials.gov (registration ID: NCT01690234). The study was approved by The Danish Regional Ethics Committee (file no: H-C-2008–112) as well as registered at and approved by the Danish Data Protection Agency.

## Background

Low back pain (LBP) is a matter of serious concern in the Western world and it is ranked as the leading non-fatal disease in the 2019 Global Burden of Disease study [1, 2]. LBP causes substantial disability among working age adults and is costly for societies with high expenses to health services, sickness absence, as well as job loss [3]. LBP is also the main reason for a premature exit from the workforce worldwide. Approximately 1.71 billion people (95% UI 1·63–1·80) have musculoskeletal conditions, with low back pain being the main health condition contributing to the need for rehabilitation services in more than 50% of the countries analysed in the Global Burden of Disease study. [4].

Recent clinical guidelines for LBP and long-term sickness absence recommended a multidisciplinary intervention in cooperation with the patient’s workplace [5–7]. Most guidelines recommend a multidisciplinary team consisting of two or more healthcare professions, i.e., medical doctors, case managers, and physical therapists [7, 8]. The evidence of the effectiveness of a multidisciplinary intervention is ambiguous. A recent Cochrane review concluded that chronic low back pain (CLBP) patients who completed a multidisciplinary rehabilitation programme, reported less pain and disability, and their chance of return to work (RTW) after 6–12 months was twice as high in comparison with usual care or exercise alone [9]. Similarly, a multidisciplinary approach has been suggested to be superior to a “single dimension” (i.e., exercise or advice) treatment in both subacute- and chronic LBP [10, 11]. With respect to RTW, several studies with various components included in multidisciplinary interventions have been conducted [12–14]. The majority of research carried out in the field of LBP focused mainly on sick-listed LBP patients [15]. The pioneering Canadian study by Loisel et al. demonstrated a positive effect of a multidisciplinary intervention focusing on gradual RTW [16]. Some of the more recent research from The Netherlands, Denmark, and Switzerland reported a positive effect of the multidisciplinary intervention on the duration of sickness absence in sick-listed LBP patients [12, 14, 17] as well as on disability and pain [14]. However, other studies were not able to demonstrate better results in terms of RTW when comparing a multidisciplinary intervention with a brief intervention [17–20].

### The Danish context

In Denmark occupied persons with LBP have 5.5 mill. days pr. year more off work when compared to occupied persons without LBP. Persons with LBP account for 1% of all somatic hospitalisations, and LBP also lead to 6% of all health-related pensions [21]. A Danish employee usually receives salary during sickness absence periods. The expenses are covered by the employer in the first 30 days. The employer can after 30 days apply for refund from the employee’s municipality to cover part of the expenses. It is possible to dismiss the employee at any time during the sickness absence. The employee on sick-leave must meet with the case-manager in the municipality sickness benefit office every 4 weeks, where rehabilitative plans are discussed [22, 23].

The health system is a public health system with free access to general practitioners, who have the gate keeper function for referral to specialist treatment.

The financial gain regarding RTW, sickness absence, and musculoskeletal disease (MSD)-related job loss from the multidisciplinary interventions appears to be limited in large community studies and workplace-based RTW-intervention studies [24]. The majority of past studies has been conducted in a clinical context, but as suggested in most guidelines such as the NICE guidelines [6], the rehabilitation process should preferably be performed in close cooperation with the patient’s workplace. Recent reviews found that multi-domain and workplace-based interventions might reduce time to RTW, reduce cost, and the cumulative duration of sickness absence in a population with varying musculoskeletal diseases [25, 26]. Furthermore, it has been suggested that a multidisciplinary intervention should be coordinated by a designated RTW-coordinator assigned to facilitate the rehabilitation- and the RTW-process [27]. However, it remains unclear how this might be organised most effectively and whether the RTW-coordinator’s professional background is of any importance. [27, 28]. It would be relevant to explore whether an early multidisciplinary intervention, involving a team of physiotherapists, ergonomists, occupational physicians, case managers, and psychologists, could render a preventive effect by retaining LBP patients, who were still working but who were at risk of deteriorated workability or of being sick-listed. When compared to the usual treatment in our institution, we anticipated a significant effect of establish contact to the workplace and the municipality sickness benefit office, but also the possibility to visit a psychologist with special competences in pain management was expected to have an effect on the outcome. In order to pursue this hypothesis, the aim of the present study was to investigate the effect of a 12-week work-oriented multidisciplinary rehabilitation programme as mentioned above coordinated by a physical therapist on the number of days off work in patients already sick-listed, or at the risk of being sick-listed, because of persistent LBP when compared to usual treatment. The secondary outcomes were back related disability, pain and quality of life.

## Methods

### Study design

A randomized controlled trial was designed, and the study protocol was performed in accordance with the relevant guidelines, the CONSORT recommendations for reporting RCT as well as the CONSORT extension for non-pharmacological treatment interventions [29, 30]. The study was registered in ClinicalTrials.gov (registration ID: NCT01690234, date 21/09/2012), and has been published [31].

### Study population

**Fig. 1:**
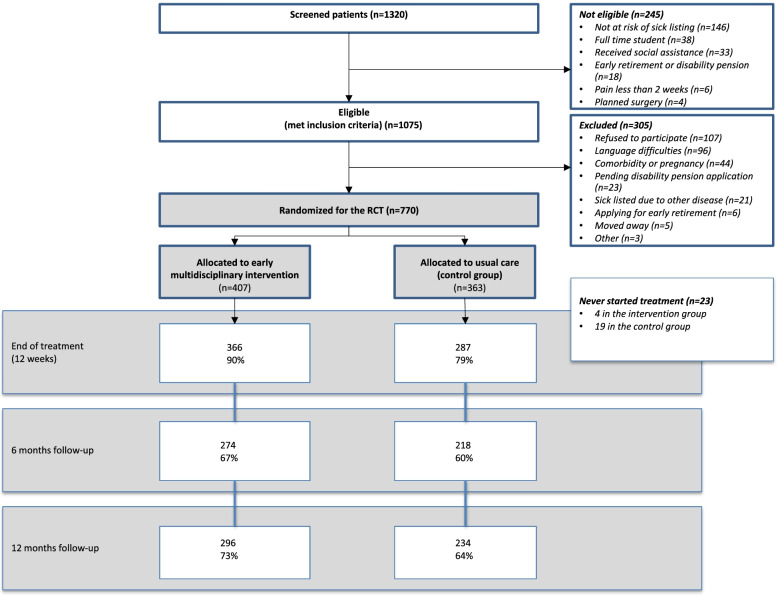
Patient flow throughout the study. Screened patients and group allocation

Among 1075 eligible patients, a total of 770 consecutive patients with LBP were enrolled in the study from September 2009 to December 2013 (Fig. 1). Patients were referred for treatment at Back Center Copenhagen, Denmark, by general practitioners, rheumatologists, or the municipal sickness benefit office.

### Criteria for inclusion and exclusion

Working age adults (18–65 years of age) with LBP for more than two weeks were eligible. The participants could be either employed or unemployed, sick-listed or at risk of being sick-listed. The not sick-listed patients were screened according to a Danish modified 17-item version of the Work Role Functioning Questionnaire (WRF-26) [32]. Patients were included if they reported being at risk of future sick-listing with at least one item scored as “100% of the time”, at least four items scored as “most of the time”, or at least eight items scored as “half of the time” [32, 33]. Exclusion criteria were red flags and comorbidity (i.e., cancer, cardiopulmonary diseases, mental diseases), pregnancy, difficulties in reading or writing the Danish language, and applicants for early retirement pension.

### Procedure of inclusion and randomization

A written information sheet was sent by post to eligible patients and was followed-up by a telephone call within two days made by a trial secretary to determine whether the patient met the inclusion criteria and was interested in participating in the study. Subsequently, a project manager included the patients at Back Center Copenhagen within one week. After signing an informed consent form, all patients filled in a baseline questionnaire (see “measurements” below). The randomization was carried out by a computer-generated list of random numbers after the patient’s completion of the inclusion interview and baseline questionnaire. A blinded independent trial secretary handled the group allocation. The patient flow throughout the study is presented in Fig. 1. Because of the nature of the treatments, it was not possible to blind neither patients nor the treatment team to the intervention. Researchers and statisticians, who obtained and analysed data, were all blinded to group assignment.

### Intervention in the usual care group

The usual care group received the standard treatment at the centre, which was to a lesser extent an interdisciplinary approach by a team including a physiotherapist or a chiropractor in cooperation with a rheumatologist and a social worker depending on the specific needs of the patient. The main focus was on a thorough physical examination, reassurance, mobilising exercises and/or motor control exercises, functional strength training, reduction of fear of movement, and guidance in self-management. The examination and treatment was based on a biopsychosocial approach according to the recent clinical guidelines [5] and the recommendations from Back Pain Europe’s evidence based guidelines [34].

### Intervention in the multidisciplinary rehabilitation group

Participants allocated to this group were met by a multidisciplinary team consisting of a “return to work coordinator”, a psychologist, an ergonomist, who gave ergonomically advice at the workplace, an occupational physician, and a case manager from the municipal sickness benefit office in addition to the usual treatment described above.

### Coordination

The treating physiotherapist served as “return-to-work coordinator” and had the first consultation with the patient. The role of the coordinator was to ensure optimal timing of the different types of intervention and facilitate the communication between all stakeholders [35]. As part of the multidisciplinary rehabilitation, patients were given an appointment with the psychologist if the patient scored above the predefined target of 1.5 on any of the 10 sub scales in SCL-90-R [36]. The main intervention strategy of the psychologist treatment was cognitive behavioural advice to address any social and personal strains and barriers related to LBP and work. If work-related disorders were suspected, a consultation with the occupational physician for assessment and advice regarding the patient’s work ability and prognosis was arranged. The workplace was involved through a workplace visit where the physical and organizational strains were evaluated by the ergonomist. A more in-depth description of the interventions is published elsewhere [31]. The patients’ case was discussed at monthly meetings with the participation of all team members. At these meetings, biopsychosocial obstacles to RTW as well as possible workplace modifications were discussed. The recommended maximal duration of treatment was 12 weeks with a maximum of 5 sessions at the psychologist. This multidisciplinary approach was added to the usual treatment (described above).

### Outcome measures

Patients in both groups filled in a questionnaire at baseline, at the end of treatment, and at a 6-month and a 12-month follow-up. A secretary with no knowledge of the group assignment sent out the follow-up questionnaires with stamped pre-addressed envelopes and entered all data in a database. The baseline questionnaires assessed demographic data, personal data, physical activity data [37], and work-related data.

### Primary outcome

The primary outcome measure was absence from the first day off work until the last day of full-time sickness absence, which was reported by questionnaire at all follow-ups. In addition, a self-reported present work status was registered.

### Secondary outcomes

The following secondary outcomes were measured: disability, measured on the Danish version of the 23-item modified Roland Morris Disability Questionnaire, RMDQ [38]. Pain intensity, measured by The Low Back Pain Rating Scale, LBP-NRS [39]; a back specific version of the Numerical Rating Scale, NRS. This scale has six 11-point subscales (range 0–10. 0 indicates no pain and 10 the worst imaginable pain.): Actual pain, worst pain in the previous two weeks and average pain in the previous two weeks as for LBP and leg pain respectively. The subscales sum up to a total pain score (range 0–60). Health related quality of life was measured by the Danish version of Short Form 36 questionnaire, SF-36 [40, 41]. SF-36 is a generic tool measuring health status with two summary scores: The Physical Component Summary (PCS) and the Mental Component Summary (MCS). Psychological distress was measured by the Danish version of The Symptom Checklist-90, SCL-90 [42]. The SCL-90 is a screening tool of general psychiatric symptomatology, which consists of 90 items, divided in 10 subscales measuring: Somatization, obsessive–compulsive, depression, anxiety, phobic anxiety, hostility, interpersonal sensitivity, paranoid ideation, psychoticism, and an additional scale concerning sleep and appetite [43]. The 90 items were scored on a five-point Likert Scale indicating the degree to which the patient was distressed by the symptom during the past week. Fear avoidance beliefs about physical activity and work were measured by the Danish version of Fear-Avoidance Beliefs Questionnaire, FABQ [44]. The questionnaire consists of 16 items to be answered on a 7-point Likert Scale. It is divided into two sub scales covering physical activity (FABQ-PA) and work (FABQ-W).

### Registration of activities

Number of consultations, referral to psychologist, occupational physician, occupational therapist, and social worker were registered together with the number of workplace visits.

### Sample size

Based on the main outcome (number of days off work) the sample size was calculated assuming an average difference between groups of 20% (5 days with an average absence period of 25 days), a dispersion of 22 days (25% of a range of 90 days), a level of statistical significance at 0.05, and a power of 0.8. Thus 305 participants were required to complete the treatment in each group. Considered a possibly high rate of withdrawal or loss to follow-up at 20% in this population, the inclusion of a total of 770 individuals was needed in the study.

### Statistical analysis

All analyses were performed according to the intention-to-treat principles using the SAS Institute 9.4 statistical software (SAS Institute Inc. 2013). To compare the effect of the two interventions on primary and secondary outcomes (between-group and repeated measurements over time), we used linear mixed models with group, gender and time as fixed variables. A mixed-effects-model was used to include information from all observations in the analysis and accounted for missing values, since missing values were missing at random. The between-group as well as the within-group effect was calculated, and outcomes were reported with 95% confidence intervals.

## Results

### Study population

A total of 770 consecutive patients were randomized to standard treatment in the usual care group (*n* = 363) or multidisciplinary intervention in the intervention group (*n* = 407) (Fig. 1). Baseline characteristics of the patients are presented in Table 1. No differences were found between the two groups in any of the baseline variables, except for a statistically significant but not clinically meaningful difference in health-related quality of life (SF-36, PCS). A drop out analysis did not reveal any statistically significant differences regarding age and gender between participants that were lost to one or more follow-ups and those that completed all follow-ups. Regarding gender there was a very small and not statistically significant difference from 52.8 for men at baseline to 49.6 at 12 months. And for women there was a difference from 47.2 at baseline to 50.4 at 12 months. For age there were no differences.

**Table 1:** Patient characteristic at baseline

**Variables**	**Intervention group (*N* = 407)**				**Usual care group (*N* = 363)**				
	N	%	Mean	SD	N	%	Mean	SD	*p*-value
**Sex (no of males)**	213	52.33			192	52.89			0.69
**Age**			39.29	9.86			39.06	10.70	0.65
**BMI**			25.70	4.83			25.65	4.36	0.92
**Current smoker**	185	46.02			166	45.98			0.96
**Marital status**									0.59
**- Married/living with partner**	258	64.02			224	62.22			
**- Living alone /divorced/widowed**	129	32.01			116	32.22			
**- Other**	16	3.97			20	5.56			
**Educational level**									0.67
**- None**	83	20.80			87	24.51			
**- Short/middle long**	275	68.92			234	65.92			
**- Long**	21	5.26			19	5.35			
**- Other**	20	5.01			15	4.23			
**Occupation**									
**- Employed**	307	75.43			292	80.44			0.62
**- Unemployed**	99	24.32			69	19.01			0.12
**Duration of pain (months)**			22.88	47.97			23.07	45.59	0.98
**Sick listed at baseline**	209	52.12			182	50.56			0.76
**Part time sick listed**	40	10.23			39	11.71			0.61
**Duration of sick listing (weeks)**			10.10	12.50			9.90	14.89	0.91
**Disability (Roland Morris 0–23) **^**a**^			14.15	4.86			14.21	4.92	0.68
**Pain (LBP rating scale 0–60) **^**b**^			28.44	11.46			29.27	10.46	0.43
**QoL (SF-36, physical 0–100) **^**c**^			49.33	8.44			50.62	8.13	0.04
**QoL (SF-36, mental 0–100) **^**d**^			50.72	10.26			49.41	10.05	0.09
**Fear avoidance (FAB-PA 0–24) **^**e**^			15.65	5.46			15.54	5.17	0.86
**Fear avoidance (FAB-W 0–42) **^**f**^			23.22	11.79			23.69	11.02	0.51
**Psychological (SCL-90) **^**g**^			0.73	0.52			0.79	0.54	0.08
**- Depression **^**h**^			1.03	0.79			1.11	0.84	
**- Somatization **^**i**^			1.11	0.58			1.19	0.59	
**Psychological total** **(above 1,5 on any sub scale SCL-90)**	178	43.7			177	48.8			0.81

^a^ Roland Morris Disability Questionnaire, RMQ

^b^ Low Back Pain Rating Scale, LBP-NRS

^c^ Short Form 36, Physical Component Summary, PCS

^d^ Short Form 36, Mental Component Summary, MCS

^e^ Fear Avoidance Beliefs Questionnaire, Physical Activity Subscale, FAB-PA

^f^ Fear Avoidance Beliefs Questionnaire, Work Subscale, FAB-W

^g^ Symptom Checklist-90, Global Severity Index, GSI

^h^ Symptom Checklist-90, Depression Subscale

^i^ Symptom Checklist-90, Somatization Subscale

### Sickness absence

There was no difference between the early coordinated group and the usual care group neither at the end of treatment or at 6 or 12 months follow-up. At baseline 53% of the patients in the intervention group and 51% in the control group were sick-listed. At the end of treatment 30% and 35% in the intervention group and usual care group, respectively, were sick listed. In both groups, 23% was sick-listed at 6-month follow-up, and 16% at 12-month follow-up (Table 2).

**Table 2:** Results of intention to treat analysis. Outcome scores and mean changes and 95% confidence intervals (CI) unless stated otherwise at the different follow-ups

	**End of treatment**				**6 months follow-up**				**12 months follow-up**			
Outcomes	Early coordinated group	Usual care group	Between group difference (95% CI)	*P*-value	Early coordinated group	Usual care group	Between group difference (95% CI)	*P*-value	Early coordinated group	Usual care group	Between group difference (95% CI)	*P*-value
Sickness absence – days off work (SD)^*^	N/A	N/A			47.52 (4.77) (38.33,56.71)	50.92 (5.26) (40.58,61.26)	3.40 (-10.43,17.24)	0.63	26.15 (3.69) (18.91,33.40)	27.15 (18.96,35.85)	1.25 (-9.87,12.38)	0.82
No. sick listed	105 (30.2%)	102 (34.7%)			60 (23.2%)	52 (23.4%)			46 (16.2%)	39 (16.2%)		
Change	-104	-102	2	0.19			8	0.23			7	0.89
Disability (RMQ) ^a^	8.36 (6.93)	7.52 (6.87)			5.80 (6.93)	5.73 (7.19)			5.77 (7.35)	5.56 (6.70)		
Change	-5.86 (-6.52,-5.19)	- 6.64 (-7.35,-5.94)	0.79 (-0.15,1.72)	0.10	-8.31 (-9.00,-7.61)	-8.53 (-9.26,-7.80)	0.23 (-0.2,1.2)	0.64	-8.43 (-9.10,-7.77)	- 8.76 (-9.46,-8.06)	0.33 (-0.6,1.2)	0.48
Pain (LBP-NRS) ^b^	18.96 (13.82)	19.93 (13.24)			18.39 (14.26)	19.48 (14.03)			17.32 (13.74)	18.69 (13.86)		
Change	-9.82 (-11.15,-8.50)	-9.24 (-10.71,-7.76)	-0.58 (-2.49,1.32)	0.55	-9.91 (-11.32,-8.49)	-9.50 (-11.10,-7.92)	-0.41 (-2.48,1.6)	0.70	-11.14 (-12.57,-9.71)	-9.96 (-11.5,-8.35)	-1.19 (-3.27,0.89)	0.26
Health related QoL (physical, PCS)^c^	59.94 (12.87)	59.81 (11.65)			60.46 (14.28)	58.64 (11.09)			63.17 (13.70)	63.09 (12.42)		
Change	10.42 (9.16,11.68)	9.55 (8.05,11.04)	0.87 (-1.05,2.80)	0.37	11.61 (9.12,14.10)	11.66 (9.17,14.16)	-0.05 (-3.56,3.45)	0.97	13.19 (11.77,14.61)	12.54 (10.91,14.17)	0.65 (-1.47,2.78)	0.55
Health related QoL (mental, MCS)^d^	52.48 (10.26)	51.46 (10.46)			53.94 (9.18)	49.94 (10.72)			54.21 (8.94)	52.92 (9.11)		
Change	2.17 (1.09,3.25)	1.79 (0.52,3.05)	0.38 (-1.18,1.94)	0.63	3.31 (0.85,5.77)	2.25 (-0.22,4.70)	1.06 (-2.34,4.47)	0.54	3.75 (2.67,4.82)	2.71 (1.50,3.92)	1.03 (-0.43;2.50)	0.17
Mental health (SCL-90)^e^ GSI	0.61 (0.60)	0.67 (0.59)			0.67 (0.71)	0.76 (0.62)			0.51 (0.55)	0.69 (0.54)		
Change	-0.12 (-0.17,-0.07)	-0.11 (-0.16,-0.05)	-0.01 (-0.09,0.06)	0.73	-0.50 (-0.18,-0.08)	-0.18 (-0.30,-0.05)	0.13 (-0.05,0.30)	0.16	-0.13 (-0.35,0.09)	-0.16 (-0.33,0.01)	0.03 (-0.24,0.31)	0.80
Depression^f^ (SCL-90 subscale)	0.87 (0.85)	0.94 (0.85)			0.88 (0.99)	1.07 (0.82)			0.68 (0.71)	0.94 (0.81)		
Change	-0.18 (-0.25,-0.10)	-0.16 (-0.24,0.08)	-0.01 (-0.12,0.01)	0.80	-0.22 (-0.42,-0.03)	-0.22 (-0.42,-0.03)	-0.001 (-0.28,0.27)	0.98	-0.33 (-0.60,-0.06)	-0.21 (-0.42,0.01)	-0.12 (-0.47,0.23)	0.49
Somatization^g^ (SCL-90 subscale)	0.89 (0.67)	0.92 (0.63)			0.96 (0.73)	1.03 (0.67)			0.96 (0.82)	1.07 (0.67)		
Change	-0.22 (-0.28,-0.17)	-0.26 (-0.32,-0.19)	0.03 (-0.05,0.12)	0.42	-0.23 (-0.37,-0.09)	-0.35 (-0.49,-0.21)	0.12 (-0.08,0.32)	0.23	-0.21 (-0.44,0.0)	-0.24 (-0.42,-0.06)	0.03 (-0.27,0.32)	0.86
Fear Avoidance, physical activity (FAB-PA)^h^	13.01 (6.38)	12.71 (5.91)			13.97 (5.93) (*n* = 39)	14.10 (5.90) (*n* = 49)			13.12 (6.70) (*n* = 17)	11.93 (6.46) (*n* = 28)		
Change	-2.28 (-3.23,-1.92)	-2.52 (-3.27,-1.76)	-0.10 (-1.02,0.90)	0.90	-1.92 (-3.40,-0.44)	-2.11 (-3.54,-0.69)	0.19 (-1.84,2.22)	0.85	-2.28 (-4.46,-0.11)	-3.64 (-5.46,-1.82)	1.34 (-1.49,4.19)	0.34
Fear avoidance, work (FAB-W) ^i^	18.97 (12.99)	19.50 (13.18)			20.65 (12.94) (*n* = 40)	21.02 (12.19) (*n* = 46)			16.81 (10.62) (*n* = 15)	17.57 (12.46) (*n* = 28)		
Change	-3.70 (-4.70,-2.71)	-2.99 (-4.14,1.89)	-0.71 (-2.22,0.79)	0.35	-1,57 (-3.89,0.75)	-5.62 (-7.99,-3.30)	4.05 (0.78,7.33)	0.02	-4.15 (-8.59,0.30)	-6.75 (-10.28,-3.21)	2.60 (-3.07,8.27)	0.36

^*^ Days off work the previous 6 months

^a^ Roland Morris Disability Questionnaire, RMQ

^b^ Low Back Pain Rating Scale, LBP-NRS

^c^ Short Form 36, Physical Component Summary, PCS

^d^ Short Form 36, Mental Component Summary, MCS

^e^ Symptom Checklist-90, Global Severity Index, GSI

^f^ Symptom Checklist-90, Depression Subscale

^g^ Symptom Checklist-90, Somatization Subscale

^h^ Fear Avoidance Beliefs Questionnaire, Physical Activity Subscale, FAB-PA

^i^ Fear Avoidance Beliefs Questionnaire, Work Subscale, FAB-W

N/A: Not applicable

–: Not measured

### Secondary outcomes

Also, disability, pain, health-related quality of life, psychological distress, and fear avoidance beliefs improved in both groups. However, no statistically significant differences were found between the groups on neither the primary nor the secondary outcomes, except for a statistically significant, but not clinically meaningful, change on FAB-W at six months.

The mean number of consultations with one or more of the team professionals was 10.7 in the intervention group and 7.1 in the usual care group. Of the 407 patients in the intervention group, 178 patients (43%) were referred to the psychologist, 45 patients (11%) to the occupational physician, 71 patients (17%) to the social worker, and 64 patients (16%) received a workplace visit. Of the 178 patients in the intervention group who were referred to the psychologist based on their score in SCL-90, only 140 (79%) in fact visited the psychologist. The reasons for not visiting the psychologist were as follows: another psychologist already treated the patient elsewhere (*n* = 5), the patient declined to consult a psychologist (*n* = 13), the multidisciplinary team in collaboration with the patient decided that the psychologist treatment was not needed (*n* = 11), or the reason was not reported (*n* = 9).

At end of the treatment, the study achieved a response rate of 86% (90% in the intervention group and 79% in the usual care group) (Fig. 1). Response rates at 6- and 12-months follow-up are presented in Fig. 1.

## Discussion

The main finding in the present study was that there were no differences between the groups at any time point for any of the outcomes. Therefore, we conclude that the intervention was not superior to usual treatment.

The lack of additional effect of our multidisciplinary intervention might be explained by several factors. Only a limited number of the patients in the intervention group received treatment and advice by the psychologist, the occupational physician, and the ergonomist. As a result, the actual treatment in the two groups might not have differed substantially. At Back Center Copenhagen, usual care already includes a limited multidisciplinary treatment focusing on the biopsychosocial factors as part of the treatment. It is possible that the usual treatment in this setting provides sufficient psychological and cognitive advice to match the patients’ needs as also suggested by Hall et al. [45]. In Denmark, the free access to treatment means that patients could simultaneously have been referred by their general practitioner to various treatments elsewhere. Even though we expected a certain level of psychosocial distress, the actual distress level was surprisingly high in the total group at baseline, and it was found to be substantially elevated compared to the general population in Denmark [46]. 46% of the study population scored above the cut-point of 1.5 at SCL-90. Psychological factors (i.e. depression, catastrophizing, and fear avoidance beliefs) have been associated with poor outcome following the treatment of LBP [47]. It is possible that the psychological guidance based on cognitive principles was not sufficiently powerful to make a difference in comparison with usual care in this sample. The amount of time spent with the psychologist might have been too limited to make an impact in these highly distressed patients as suggested by Pincus et al. [47]. In both treatment groups, the percentage of sick-listed patients decreased from about 50% at baseline to 16% at 12-month follow up. These results are similar to those reported by other studies in European settings [12, 17]. It is unclear whether this decline would occur even without treatment due to natural history.

The effectiveness of multidisciplinary interventions for RTW outcomes is debatable. A recent systematic review provided moderate quality evidence that multidisciplinary rehabilitation involving a physical component and or both a psychological component and a social/work targeted component, appears to be more effective than physical treatment but not more effective than usual care in patients with CLBP [9]. A closer look exclusively at randomized studies comprising multidisciplinary rehabilitation with a Cognitive Behavioural Therapy (CBT) approach that include workplace intervention reveals an unclear pattern with respect to work participation at 12 months follow-up. Our results were in concordance with the results of other Nordic studies reporting no benefit when multidisciplinary rehabilitation including workplace intervention was compared with care managed by the general practitioner [48, 49] or mini-intervention [17, 20].

However, our results appear not to be in concurrence with those of other studies reporting benefits when the effect is compared with that of multidisciplinary intervention without workplace involvement [12, 16] or exercise therapy [50]. Two additional studies reported benefits if the effect is compared to that of graded activity including workplace involvement [51] or workplace involvement alone [52].

Our study population comprised sick-listed as well as not-sick-listed participants. Thus, a comparison with studies that merely included sick-listed patients should be made with caution. To our knowledge, only one randomised study by Jensen et al. has been published including a patient sample similar to the present population [53]. Even though the study populations are to some extent comparable, the interventions and results from the two studies differ. The intervention in Jensen’s study was brief and consisted mainly of two consultations by an occupational physician focusing on workplace barriers and advice to stay active. The authors observed a positive effect in pain and physical function, suggesting that these two domains were the key components. Others have suggested that the number of disciplines involved in the multidisciplinary rehabilitation contributed to the outcome with respect to work participation [11]. However, given the broad range of professions in the rehabilitation team, this hypothesis was not supported by our study.

### Strengths and limitations

The main strengths of our study were the randomized study design, the usual care group resembling daily practice, the large sample size, and that the intervention was carried out in a practice-oriented setting in already existing organisations. Several limitations to the study design may have influenced the results of this study. Firstly, 23 patients (4 and 19 in the intervention and usual care groups respectively) did not participate in the full treatment programme. The response rate at end of the treatment was 90% in the intervention group and 79% in the usual care group. At 6-month follow-up, 68% (67% and 60% in the intervention and usual care groups, respectively) completed the 6-month follow-up, and 71% (73% and 64% in the intervention and usual care groups, respectively) completed the 12-month follow-up. It is not clear whether this difference in dropouts has led to an under- or an overestimation of the differences between the groups. However, inasmuch as we used mixed models that yield unbiased estimates for this scenario, where data were missing at random, a possible differential dropout bias is accounted for by this method [54]. At 6-month follow-up, 55 participants had not yet completed their course of treatment and consequently, the end of treatment and the 6-month follow-up questionnaires were identical, meaning that the actual response rate after 6-month was 73%. Due to dropouts during the study period the actual no of patients at the end, the study may have been underpowered. Most importantly, only a reduced number of patients with a high score on SCL attended a psychologist, meaning that a washout effect of this part of the intervention is likely. A similar concern applies to the limited number of workplace visits. This was in fact a deviation from the study protocol. Secondly, although we intended to use both self-reported data and data from a public data base on sickness absence when we designed the study, it was not possible to get the data from the data-base after finalising the study. This was also a deviation from the study protocol. Thirdly, some degree of contamination might have taken place since as both groups attended the same centre, and the practitioners might have inspired each other unintentional regarding the multidisciplinary treatment approach. Finally, the team had experienced some difficulties managing the intervention: several of the practitioners were replaced during the study, the period between meetings might have been too long, not all practitioners were present at all meetings, and on some occasions the coordinators had difficulties in carrying out decisions on behalf of the team resulting in a lack of timeliness and continuity.

The intention when designing the study was to test an early intervention, but in reality, 47% of the patients had a symptom duration of more than 3 months, and the average time off work among the sick-listed patients was 10 weeks. This unexpected level of chronicity might be explained by the fact that this study was conducted in already existing organisations, and standard referral procedures from the GPs to the centre required that patients had undergone mono-disciplinary treatment in primary care without a satisfactory outcome.

## Conclusion

In this study, we found that a multidisciplinary intervention had no additional effect compared to usual care on any of the outcomes: sickness absence, disability, pain, or health related quality of life.

## Data Availability

The data that support the findings of this study are not publicly available due to the sensitivity of human data but are available from the corresponding author upon reasonable request. Contact should be made via the corresponding author, Annette Fisker fiskerannette@gmail.com.
